# Editorial: Design, synthesis, fundamental understanding, and advanced applications of molecular aggregates

**DOI:** 10.3389/fchem.2022.957621

**Published:** 2022-08-25

**Authors:** Yuncong Chen, Haoke Zhang, Zijie Qiu, Ming Chen, Dong Wang

**Affiliations:** ^1^ State Key Laboratory of Coordination Chemistry, School of Chemistry and Chemical Engineering, Chemistry and Biomedicine Innovation Center (ChemBIC), Nanjing University, Nanjing, China; ^2^ MOE Key Laboratory of Macromolecular Synthesis of Functionalization, Department of Polymer Science and Engineering, Zhejiang University, Hangzhou, China; ^3^ School of Science and Engineering, Shenzhen Institute of Aggregate Science and Technology, The Chinese University of Hong Kong, Guangzhou, China; ^4^ College of Chemistry and Materials Science, Jinan University, Guangzhou, China; ^5^ Center for AIE Research, Shenzhen Key Laboratory of Polymer Science and Technology, Guangdong Research Center for Interfacial Engineering of Functional Materials, College of Materials Science and Engineering, Shenzhen University, Shenzhen, China

**Keywords:** AIE (aggregation induced emission), aggregate science, bioimaging, theranostics, AIEgens

Research on aggregation-induced emission (AIE) has attracted much attention since the concept of AIE was coined in 2001 (Liu et al., 2022). Aggregation-induced emission luminogens (AIEgens) have emerged as a new type of advanced function materials with various practical applications (Yang et al., 2020). After over 20 years, AIE research has been extended to aggregate science, which showed a much wider scope not limited to emission related research fields, such as polymers, mixtures, nanoparticles, metal-organic frameworks, supramolecular self-assembly, stimulus response systems, clean energy, optoelectronic devices, artificial photosynthesis, photovoltaic cells, light-emitting materials, chemical sensing, biological probes, biomedical imaging, diagnosis and treatment, drug delivery, and many other frontier fields. This Research Topic aims to collect recent progress in the design and synthesis, fundamental understanding, and advanced applications of molecular aggregates. The topic includes one original research article, one mini review, and two reviews.

Biosensing and bioimaging represent important applications of molecular aggregates, in which enhanced emission can be achieved upon the formation of molecular aggregates or binding molecules with biomolecules in the surrounding bio-environment. The collaborative study led by Pengfei Zhang et al. explores a double-checked Logic Gate system comprised of both CRISPR/Cas12a and DNA-binding AIE molecule for the detection of pathogenic microorganisms. The presented AIE molecules are non-emissive in an aqueous solution but emit intensively in the presence of a large amount of DNA produced by recombinase polymerase amplification (RPA) within a short time period, with an extremely high targeting-specificity via a restriction of intramolecular motion mechanism. This finding is useful for detecting pathogenic microorganisms in environmental water samples.

Cheng et al. reviewed the 3D fibrous aerogels for solar vapor generation (SVG) based on building block synthesis, photothermal materials selection, porous structure construction, and device design. In this study building blocks based on natural cellulose nanofibers, petrochemical polymer nanofibers, and inorganic nanofibers were introduced to fabricate aerogels. Plasmonic nanometals, semiconductors, carbonaceous polymers, MXene, and AIE molecules are selected as photothermal materials. To improve the evaporation efficiency, the porous structures of these building blocks and photothermal materials are crucial factors. For one of the applications, water purification, the SVG-based technology shows advantages for both large-scale and personal use with inexhaustible solar energy, independent of electricity and complex infrastructure. At the end of the review, they propose three strategies to further promote SVG utility, such as tailoring the hydrophilicity of the fibrous building blocks to increase the interaction with bonded water, multifunctional SVG with anti-biofouling activity, photocatalysis, and energy transformation property, exploring simple fabrication methods and low-cost starting fiber materials.

A new class of AIEgens of racemic C6-unsubstituted tetrahydropyrimidines (THPs) are reviewed by Zhu. Interestingly, unlike the conventional AIEgens with conjugated aromatic structure, they possess a non-aromatic chiral central ring (tetrahydropyrimidine), which is attached by three completely non-crowded and freely rotatable phenyl rings. Although non-successive conjugation is found in these AIEgens, the through-space conjugation between the isolated phenyl rings can induce a remarkable emission in the visible light region. Their molecular conformation is flexible and thus, the RIM can be explained by the AIE behavior. Moreover, the AIEgens can be readily obtained with different substituents which allow for tuning the AIE effect, emission wavelength, luminescent efficiency (up to 100%), packing model, and polymorph in the aggregate state. The collective properties enable them to be competent in the applications of critical micelle concentration (CMC) fluorescent detection, long-term cell imaging, fluorescence on-off thermometer, mechanofluorochromism, and copper (II) probe. This unusual structure with interesting properties will likely inspire more studies in the future.

Zheng et al. review recent works investigating the structure-property relationships of organic molecular aggregates in different environments, including crystal, co-crystal, amorphous aggregate, and doped systems, by the multiscale modeling protocol. Different molecular models were summarized according to the relevant micro-environments: 1) the implicit solvation model was used to consider the solvent effect; 2) the hybrid quantum mechanics/molecular mechanics (QM/MM) model was chosen to consider the influence of molecular packing on photophysical properties; 3) molecular dynamics simulation was applied for the complicated conformations of amorphous aggregates. This work systematically discussed the influence of intermolecular noncovalent interactions on molecular packing and their photophysical properties. It could therefore provide powerful guidelines for the design, synthesis, and development of advanced organic luminescent materials.
